# Presumed Cerebral Vasculitis Following Pneumococcal Meningitis: A Rare but Life-Threatening Complication

**DOI:** 10.1155/carm/9029598

**Published:** 2025-08-22

**Authors:** Daniel Matassa, Pooja Patel, Lisa Dever

**Affiliations:** ^1^Department of Medicine, Rutgers New Jersey Medical School, Newark, New Jersey, USA; ^2^Department of Neurology, Rutgers Robert Wood Johnson Medical School, New Brunswick, New Jersey, USA

## Abstract

A 65-year-old woman presented with pneumococcal sepsis and meningitis. Despite appropriate antimicrobial therapy and intravenous (IV) dexamethasone, her mental status did not improve. Findings of brain imaging were suggestive of cerebral vasculitis. Her condition improved rapidly with the initiation of high-dose IV methylprednisolone therapy. One week after completion of a 3-week oral prednisone taper, the patient's neurocognitive deficits recurred. A more extensive diagnostic evaluation, including conventional angiography, was again consistent with a presumptive diagnosis of cerebral vasculitis. High-dose IV methylprednisolone and a more prolonged taper of oral prednisone led to remission of her disease, with marked improvement in mental status and left-sided hemiparesis. Cerebral vasculitis is a rare but potentially lethal complication of pneumococcal meningitis that may be difficult to recognize and treat. The role of brain biopsy in diagnosis is unclear, and due to its rarity, management of this condition lacks conclusive evidence-based data.

## 1. Background

*Streptococcus pneumoniae* is the most common cause of bacterial meningitis in adults [1]. Even when treated promptly and appropriately, pneumococcal meningitis has been associated with neurological complications, including seizures, cerebral edema, ischemic and hemorrhagic stroke, hydrocephalus, and rarely, cerebral vasculitis [2]. We describe an unusual presentation of recurrent cerebral vasculitis and encephalopathy following pneumococcal meningitis that responded to high-dose prolonged corticosteroid therapy.

## 2. Case Presentation

**Figure 1:**
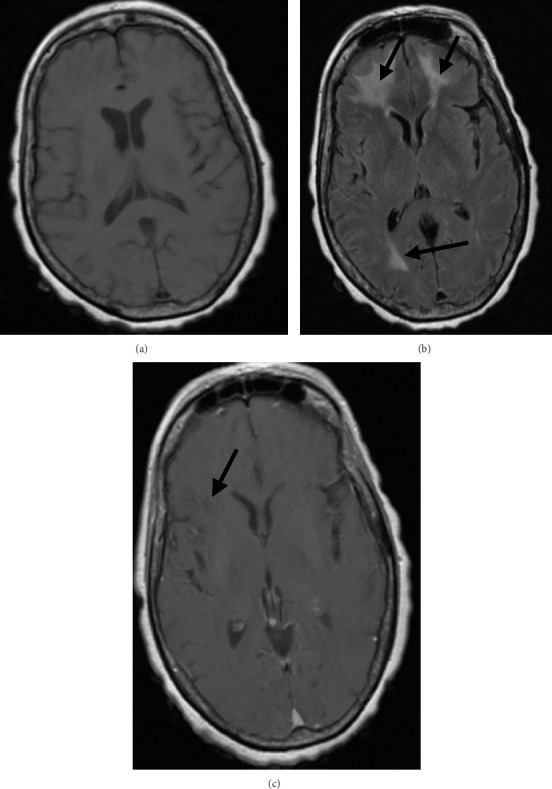
MRI brain, with and without contrast—hospital admission #1. Abnormal findings are indicated by the arrows. (a) Axial T1—no significant findings. (b) Axial T2 FLAIR-increased signal abnormality in the bilateral frontal and right occipital lobes. (c) Axial T1 postcontrast—faint linear enhancement of the white matter parenchyma in the bilateral frontal lobes (greater on the right). Altogether, this MRI study demonstrates diffuse areas of white matter enhancement, vasogenic edema, and diffusion restriction consistent with a cerebral vasculitis within the parietal and frontal lobes.

A 65-year-old woman with no known significant past medical history presented to the emergency department with fever, chills, fatigue, and altered mental status that progressed over two days. Her vital signs were normal with the exception of a temperature of 101.8°F. She was acutely ill, obtunded, noncommunicative, and unable to follow commands. She had nuchal rigidity but no overt focal neurologic deficits. Laboratory evaluation was remarkable for a white blood cell count of 9100 cells/μL with 14% band, platelets of 78,000/μL, an elevated lactic acid of 5.4 mmol/L, and mild elevations in transaminases. INR and fibrinogen levels were normal. Chest radiograph was normal. Computed tomography (CT) scan of the head demonstrated no acute pathology. Intravenous (IV) dexamethasone and empiric broad-spectrum antimicrobials (ampicillin, vancomycin, cefepime, and acyclovir) were administered. Cerebrospinal fluid (CSF) analysis demonstrated a neutrophilic pleocytosis, hypoglycorrhachia, and Gram-positive cocci in pairs and chains. Blood and CSF cultures grew pan-susceptible *Streptococcus pneumoniae*, and the antibiotic regimen was de-escalated to ceftriaxone alone (Table 1). The patient's sepsis resolved after 48 h of therapy with antimicrobials, but she had minimal improvement in mental status. Video electroencephalogram (vEEG) demonstrated left frontal focal seizures, with frequent left central sharp waves. Despite treatment with levetiracetam and resolution of seizures on vEEG, her mental status was unchanged. A therapeutic lumbar puncture was repeated on hospital day three, with an opening pressure of 27.5 cmH_2_0 and 23.5 mL of CSF removed. CSF analysis demonstrated an improvement in protein and glucose to 208 mg/dL and 41 mg/dL, respectively. Magnetic resonance imaging (MRI) of the brain performed on hospital day five showed diffuse areas of white matter contrast enhancement, vasogenic edema, and diffusion restriction, suggestive of a cerebral vasculitis within the parietal and frontal lobes (Figure 1). A CT angiogram of the head and neck demonstrated diffuse white matter hypodensities, but it was not sensitive enough to detect medium-to-small vessel changes. With a possible diagnosis of cerebral vasculitis based on imaging findings, on hospital day 5, the patient was treated empirically with IV methylprednisolone 1 g every 24 h with prompt improvement in her mental status. This improvement allowed for a more thorough neurologic exam, which demonstrated left-sided hemiparesis. Digital subtraction angiogram was deferred given rapid improvement in her clinical condition. After 5 days of high-dose methylprednisolone, she was transitioned to oral prednisone 80 mg every 24 h with tapering over 3 weeks in decrements of 20 mg every 5 days. At the time of discharge to subacute rehabilitation, the patient was alert and oriented to person, place, and time, and was able to follow commands and communicate in short sentences. She was scheduled to complete 2 weeks of IV ceftriaxone and the remainder of her 3-week oral prednisone taper.

One month following discharge, the patient was readmitted with a 1-week deterioration of mental status following discontinuation of prednisone. She had progressive lethargy and confusion and was noncommunicative. She was afebrile and had normal vital signs. She was nonverbal, unable to follow commands, responsive to tactile stimulation, and withdrawing from pain in bilateral upper and lower extremities. Laboratory results were significant for hyponatremia with sodium 126 mEq/L and an elevated erythrocyte sedimentation rate (ESR) of 43 mm/hr and C-reactive protein (CRP) of 12 μg/mL. Chest radiography and CT of her head were normal.

**Figure 2:**
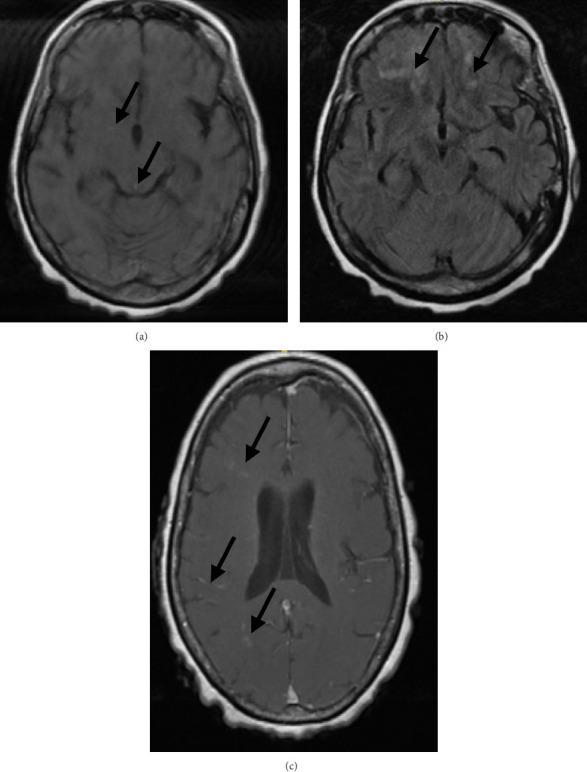
MRI brain, with and without contrast—hospital admission #2. Motion artifact limited some images. Abnormal findings are indicated by the arrows. (a) Axial T1-acute lacunar infarcts and microhemorrhages in right putamen and right inferolateral tectum. (b) Axial T2 FLAIR—decreased FLAIR signal compared to prior image, in the bilateral frontal lobes. (c) Axial T1 postcontrast-increased enhancement in right frontal, parietal, and occipital lobes, compared to prior study. Altogether, this MRI study demonstrates scattered acute lacunar infarcts, subacute cerebral infarcts and microhemorrhages, and an increase in contrast enhancement, suggestive of active and progressive cerebral vasculitis.

Given the concern for recurrent meningitis and relapse of cerebral vasculitis, the patient was treated with empiric IV vancomycin, ceftriaxone, and methylprednisolone. vEEG demonstrated a left frontal epileptogenic focus with moderate background slowing, as noted on prior vEEG, without evidence of ongoing clinical or subclinical seizures. A lumbar puncture was performed. CSF analysis demonstrated mildly elevated protein (46 mg/dL) but was otherwise normal. Blood and CSF cultures were negative. Diagnostic studies for paraneoplastic and infectious etiologies were negative. MRI of the brain demonstrated scattered acute lacunar infarcts, subacute cerebral infarcts and microhemorrhages, and an increase in contrast enhancement, suggestive of active and progressive cerebral vasculitis (Figure 2).

Antibiotics were discontinued, and pulse-dose IV methylprednisolone (1 g every 24 h) initiated for treatment for relapsed CNS vasculitis. Transesophageal echocardiogram was normal, as was telemetry monitoring. Rheumatoid factor was positive, but all other rheumatological diagnostic tests including complement levels, anti-SSA/SSB, anti-CCP, ANA, ANCA, and antiphospholipid antibody levels were normal.

**Figure 3:**
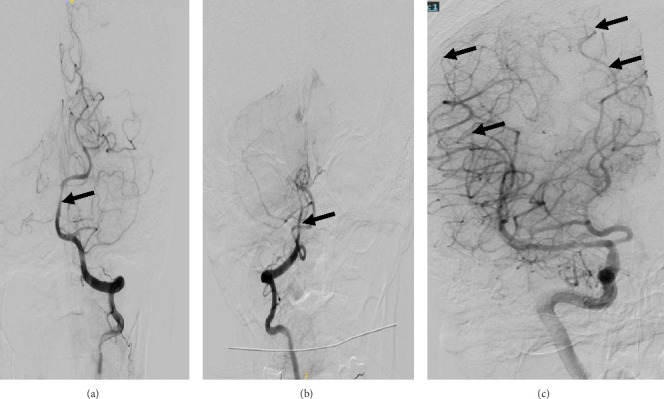
Digital subtraction angiography—hospital admission #2. Abnormal findings are indicated by the arrows. (a) and (b) Left and right vertebral arteries, respectively, showing distal basilar artery vessel irregularity. (c) Right internal carotid artery and its territories, showing diffuse vessel irregularities in the medium to small branches of the right middle cerebral artery.

*Digital* subtraction angiography (DSA) demonstrated significant vessel irregularities in the distal basilar artery and medium to small branches of the right middle cerebral artery, as well as a right cerebral arteriovenous malformation (AVM). The brain lesions on cerebral imaging and the appearance of the vessels on DSA were most consistent with a diagnosis of cerebral vasculitis, and deemed reversible cerebral vasoconstriction syndrome to be a less likely cause (Figure 3). Diagnostic leptomeningeal biopsy was considered, but given the invasiveness of the procedure and the patient's clinical improvement, the family declined.

The patient received 3 days of IV methylprednisolone with marked improvement in mental status, and improvement in left-sided upper and lower extremity weakness. She was transitioned to oral prednisone 80 mg daily and discharged to a subacute rehabilitation facility. Her prednisone was tapered over 3 months. Rheumatology recommended treatment with cyclophosphamide or rituximab if the patient relapsed after discontinuation of prednisone.

The patient's condition continued to improve with resolution of left-sided extremity weakness. Further immunosuppression was deemed unnecessary. She subsequently underwent successful stereotactic radiosurgery for her AVM. She had no recurrence of neurological symptoms 1 year after discontinuation of prednisone but has mild residual memory impairment.

## 3. Discussion

*Streptococcus pneumoniae* is the most common cause of bacterial meningitis in the adult population [1]. Despite the introduction of the pneumococcal conjugate vaccines and increased rates of vaccination, the incidence of pneumococcal meningitis in adult patients has not changed [3, 4]. Infection of the meninges results in a significant inflammatory response with an influx of neutrophils and inflammatory cytokines, often resulting in neurological sequelae [1]. While the acute morbidity and mortality of pneumococcal meningitis, and the need for timely evaluation and treatment, are well recognized, clinicians may not be aware of serious neurological and immunological sequelae that can occur even after successful antibiotic treatment. Reported sequelae include seizures, cerebral edema, hemorrhagic or ischemic stroke, hydrocephalus, and cerebral vasculitis [2]. Cerebral vasculitis is often categorized as either primary angiitis of the central nervous system (PACNS) or as secondary to a systemic vasculitis or infection and is extremely rare, with an incidence rate of 2.4 per million [5]. Many infectious pathogens have been linked to the development of cerebral vasculitis, including *S. pneumoniae*, with molecular mimicry the predominant mechanism reported [6]. There are few case reports of pneumococcal meningitis complicated by presumed cerebral vasculitis [7–9]. Our case provides additional information to guide the diagnosis and management of presumed cerebral vasculitis following pneumococcal meningitis.

Prompt recognition and management of cerebral vasculitis can be challenging given the rarity of the condition and the fact that there are several differential diagnoses that can imitate it. Mimicking diseases, such as RCVS and reversible posterior leukoencephalopathy syndrome (RPLS), share many of the same clinical and imaging features of CNS vasculitis. Subtle differences in the clinical course or diagnostic imaging, and the findings of advanced neurovascular studies and biopsies, can help the clinician to select and treat the most plausible diagnosis—but the findings are rarely straightforward and can often be misleading.

The disease presents with a variety of radiographic and clinical findings dependent on the cerebral vasculature affected. MRI findings of the brain often include bilateral hyperintense and gadolinium-enhancing lesions of the cerebral cortex, white matter, and meninges, as well as small ischemic and hemorrhagic strokes [10]. CT and MR angiographic imaging studies have a limited role in the diagnosis of this disease. Black blood vessel wall imaging is an MRI technique that is gaining increasing recognition in the diagnosis of many intracranial vasculopathies, but further studies and guidelines are needed to elucidate its exact role in the diagnostic work-up of cerebral vasculitis [11]. CSF analyses often demonstrate normal glucose and elevated protein levels [12], findings that are nonspecific. Advanced evaluation of cerebral vasculitis requires conventional angiography, with most experts recommending a brain and leptomeningeal biopsy [13–15]. DSA demonstrates segmental narrowing of small- to medium-sized arteries, in multiple vascular territories, often with circumferential or eccentric vessel irregularities—findings which can be mimicked by multiple other cerebrovascular disorders [16]. Histopathology demonstrates granulomatous angiitis of the small- to medium-sized arteries in half of cases, with the other half composed of lymphocytic vasculitis [13]. Although biopsy is considered a gold standard by many experts—to both confirm cerebral vasculitis and rule out mimicking differential diagnoses—the procedure is invasive, and the false negative rate is estimated to be 25%, with a suboptimal diagnostic accuracy between 64% and 84.1% [14, 15].

The diagnosis of cerebral vasculitis, as proposed in 1988, requires the presence of 3 clinical criteria: (a) an acquired, otherwise unexplainable neurological deficit; (b) evidence of classic angiographic or histopathologic features of angiitis within the CNS; and (c) no evidence of a systemic vasculitis or any other condition that could mimic the radiographic/pathologic findings [17]. Our patient technically met these diagnostic criteria with neurocognitive deficits—without other clear identifiable causes on extensive serologic and radiographic diagnostic evaluation—and angiographic findings consistent with the diagnosis. Additionally, her overall clinical trajectory and response to treatment were most consistent with cerebral vasculitis, even without a biopsy. Her successful clinical outcome challenges current expert opinion that all patients with suspected cerebral vasculitis undergo brain biopsy. We propose that in certain clinical scenarios, such as this one where there was a clear sequence of events and supportive imaging and angiographic data, an invasive brain biopsy could be foregone. The 1988 criteria, which do not require a pathology result to make the diagnosis, are supported by cases such as ours. However, we do believe that with discrepancies in expert opinion, aging guidelines, and significant advances in neuroimaging, revised criteria are needed to assist in diagnosing cerebral vasculitis.

Our case also demonstrates the lack of consensus for corticosteroid dosing in the treatment of cerebral vasculitis. Our patient was initially treated with a 5-day course of pulse IV methylprednisolone and an oral taper of prednisone over 3 weeks. Following completion of the taper, she had deterioration in mental status. After treatment with high-dose IV methylprednisolone, she was discharged with a 3-month oral prednisone that was successful in preventing recurrence. There are no clinical studies identifying the optimal corticosteroid agent, dosing, and duration. In addition, the decision to use cyclophosphamide and/or rituximab in the induction or maintenance treatment of these cases is primarily based on studies and anecdotal evidence from other rheumatologic conditions. Despite current recommendations favoring the usage of cyclophosphamide in cerebral vasculitis, there is a lack of data on the efficacy of rituximab as an alternative. Given the patient's improvement with prolonged corticosteroid therapy, the decision was made to forego additional immunosuppression. Our case highlights the need for additional data on the optimal management of cerebral vasculitis in the context of bacterial meningitis, including the need for brain and leptomeningeal biopsy, dosing and duration of corticosteroid therapy, and the role of advanced immunosuppressives.

## Figures and Tables

**Table 1:** Blood culture and CSF results from initial admission (normal range).

CSF opening pressure	20 cm H_2_0
CSF glucose	< 2 mg/dL (40–80 mg/dL)
CSF protein	> 600 mg/dL (15–45 mg/dL)
CSF lactic acid	23.1 mmol/L (0–2.2 mmol/L)
CSF white blood cells	41 c/μL (69% neutrophils, 20% lymphocytes)
CSF red blood cells	24 c/μL
CSF culture	*Streptococcus pneumoniae*–susceptible to ceftriaxone, clindamycin, erythromycin, levofloxacin, penicillin, tetracycline, trimethoprim/sulfamethoxazole
Blood cultures	4 bottles positive for *Streptococcus pneumoniae* with the same susceptibility results

## Data Availability

Data sharing is not applicable as no new data were generated.
